# p53 promotes the expression of gluconeogenesis-related genes and enhances hepatic glucose production

**DOI:** 10.1186/2049-3002-1-9

**Published:** 2013-02-04

**Authors:** Ido Goldstein, Keren Yizhak, Shalom Madar, Naomi Goldfinger, Eytan Ruppin, Varda Rotter

**Affiliations:** 1 Department of Molecular Cell Biology, Weizmann Institute of Science, 234 Herzl Street, Rehovot, 76100, Israel; 2 Blavatnik School of Computer Science, Tel Aviv University, Haim Levanon, Tel Aviv, 69978, Israel; 3 Sackler School of Medicine, Tel Aviv University, Haim Levanon, Tel Aviv, 69978, Israel

**Keywords:** p53, Gluconeogenesis, Hepatic glucose production, Diabetes

## Abstract

**Background:**

The p53 tumor suppressor protein is a transcription factor that initiates transcriptional programs aimed at inhibiting carcinogenesis. p53 represses metabolic pathways that support tumor development (such as glycolysis and the pentose phosphate pathway (PPP)) and enhances metabolic pathways that are considered counter-tumorigenic such as fatty acid oxidation.

**Findings:**

In an attempt to comprehensively define metabolic pathways regulated by p53, we performed two consecutive high-throughput analyses in human liver-derived cells with varying p53 statuses. A gene expression microarray screen followed by constraint-based modeling (CBM) predicting metabolic changes imposed by the transcriptomic changes suggested a role for p53 in enhancing gluconeogenesis (*de novo* synthesis of glucose). Examining glucogenic gene expression revealed a p53-dependent induction of genes involved in both gluconeogenesis (*G6PC*, *PCK2*) and in supplying glucogenic precursors (glycerol kinase (GK), aquaporin 3 (AQP3), aquaporin 9 (AQP9) and glutamic-oxaloacetic transaminase 1 (GOT1)). Accordingly, p53 augmented hepatic glucose production (HGP) in both human liver cells and primary mouse hepatocytes.

**Conclusions:**

These findings portray p53 as a novel regulator of glucose production. By facilitating glucose export, p53 may prevent it from being shunted to pro-cancerous pathways such as glycolysis and the PPP. Thus, our findings suggest a metabolic pathway through which p53 may inhibit tumorigenesis.

## Findings

p53 is a transcription factor that regulates the expression of many genes, thereby eliciting a myriad of cellular responses, most of them culminate in an anti-tumorigenic effect [1]. Recently, the concept of p53 as a regulator of metabolism has emerged with various metabolic pathways found to be regulated by p53 in an anti-tumorigenic effort [2,3].

**Figure 1:**
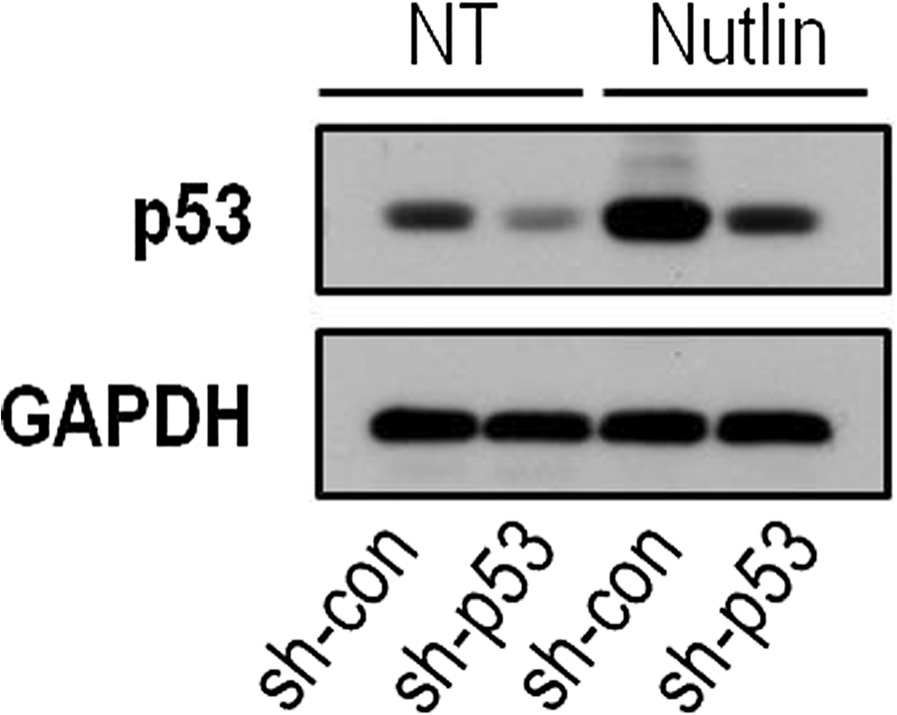
**p53 protein level is elevated in HepG2 cells following Nutlin-3a treatment**. HepG2 cells were treated with the p53-activating agent Nutlin-3a (10 μM) for 24 hours, total protein was extracted and the protein level of p53 was analyzed in a western blot analysis (as described in [13]). The presented data is a representative experiment from more than three repeats.

To comprehensively describe the effect of p53 on liver-related metabolic pathways, we performed two consecutive high-throughput analyses. We evaluated the global alterations in gene expression in liver-derived HepG2 cells following two reciprocal manipulations in p53 status. First, we down-regulated p53 levels in a group of HepG2 cells by stably expressing short hairpin RNA targeting p53 (termed HepG2^sh-p53^) while the control group expressed a non-relevant short hairpin RNA (termed HepG2^sh-con^). Second, we treated the cells with Nutlin-3a, a p53-activating agent, resulting in accumulation of p53 protein levels and induction of p53-dependent transcriptional programs (Figure 1) [4]. The transcriptome of HepG2 cells under the four different conditions was analyzed using gene expression microarrays (as described in [4]). Next, we analyzed the way these p53-dependent changes in the transcriptome are predicted to affect metabolic pathways. This was done by constraint-based modeling (CBM), a widely used computational method for studying metabolism on a genome-scale, which has been successfully used for a variety of applications (Additional file 1 and [5]). Recently, two models of human metabolism were published and their potential clinical utility has been demonstrated [6,7]. The generic human models have also served as a basis for generating context-specific models including liver metabolic activities [8] and cancer metabolism [9]. We utilized a CBM method termed integrative metabolic analysis tool (iMAT), which integrates the gene expression levels measured under different conditions to predict a most-likely distribution of metabolic fluxes, accounting for post-transcriptional flux activity predicted via stochiometric considerations [10,11]. We applied iMAT to Nutlin-treated HepG2^sh-con^ cells versus Nutlin-treated HepG2^sh-p53^ cells. Performing pathway enrichment analysis over the set of reactions predicted to be active in each state, we found a significant enrichment of metabolic reactions associated with gluconeogenesis in the Nutlin-treated HepG2^sh-con^ cells (*P* = 0.001, after correcting for multiple hypothesis using false discovery rate (FDR) with α = 0.05), while no enrichment was found for these pathways in the Nutlin-treated HepG2^sh-p53^ cells (for methods summary see Additional file 2). In addition to gluconeogenesis, reactions in the fatty acid oxidation pathway were also predicted to be active in p53-expressing cells. This pathway was previously shown to be induced by p53 thus attesting to the validity of our analysis [12]. A full list of reactions predicted to be active or inactive following alterations in p53 status is presented in Additional file 2: Table S1.

**Figure 2:**
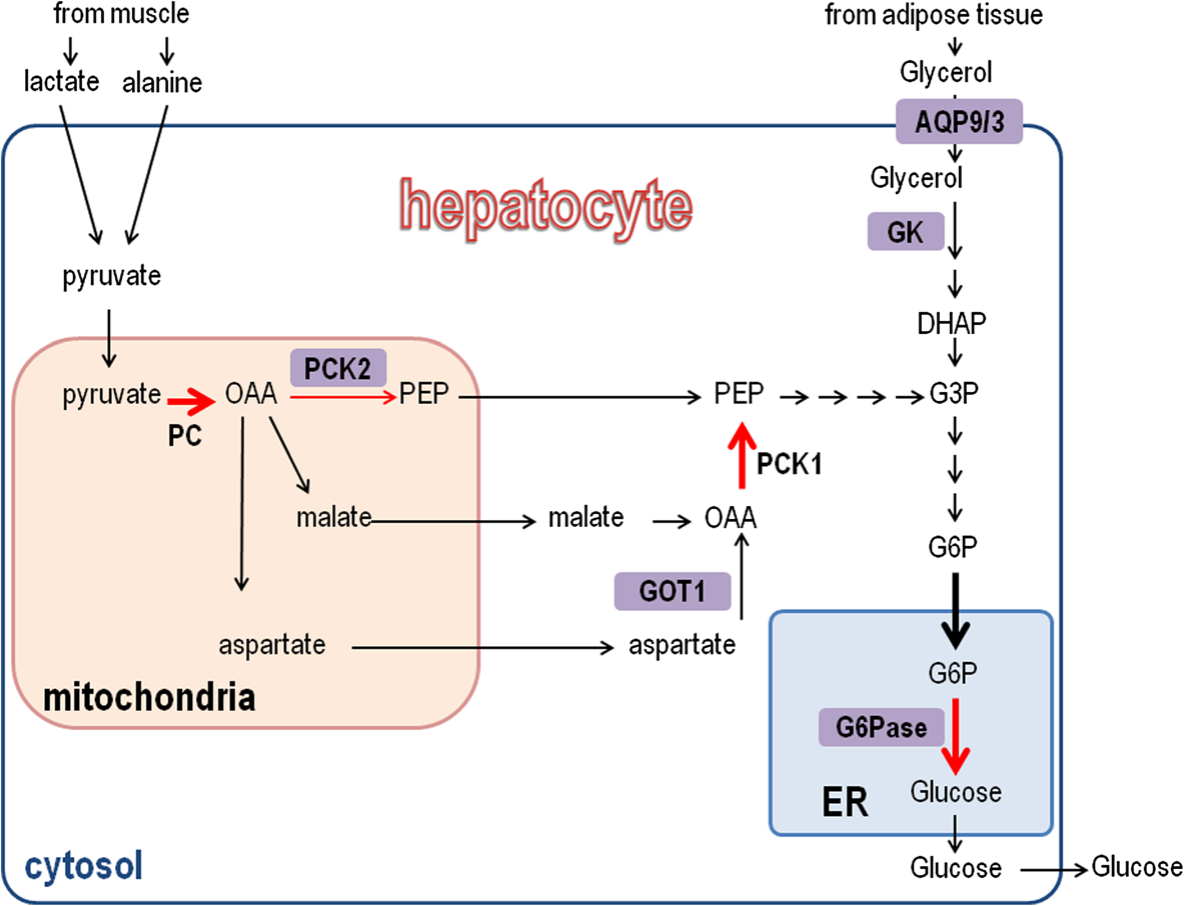
**Activation of p53 is predicted to increase the flux through gluconeogenesis.** The pathways supplying precursors to gluconeogenesis and the glucogenic pathway are depicted. Red arrows represent the transcriptionally-regulated reactions of gluconeogenesis. Bold arrows represent reactions predicted to carry higher flux in p53-expressing cells according to iMAT. Proteins whose genes are induced by p53 are depicted in purple rectangles. DHAP, dihydroxyacetone phosphate; G3P, glyceraldehyde 3-phosphate, G6P, glucose-6-phosphate, iMAT, integrative metabolic analysis tool; OAA, oxaloacetate; PEP, phosphoenolpyruvate.

Our analyses pointed to a role for p53 in regulating gluconeogenesis (*de novo* synthesis of glucose). Gluconeogenesis is activated in hepatocytes when blood glucose levels diminish (due to fasting or vigorous exercise), the glucose is then secreted to the bloodstream thereby restoring glucose homeostasis. Upon a hormonal stimulus the induction of gluconeogenesis is achieved through transcriptional, post-translational and allosteric alterations in four main reactions of gluconeogenesis. These reactions are catalyzed by the following enzymes: pyruvate carboxylase (PC), phosphoenolpyruvate carboxykinase (PEPCK), fructose-1,6-bisphosphatase (F1,6BPase) and glucose-6-phosphatase (G6Pase). Transcriptional regulation of the genes encoding these enzymes is crucial for an effective induction of gluconeogenesis [14]. Concomitantly with these alterations, the liver is supplied with glucogenic precursors from extra-hepatic tissues, which are converted to pyruvate and enter the glucogenic pathway. The main precursors are lactate and alanine (arriving from muscles during exercise) and glycerol (arriving from adipocytes during lypolysis) (Figure 2).

Focusing on reactions associated with the gluconeogenesis pathway, we next evaluated the predicted flux rate carried by each of these reactions in each condition. Strikingly, three of the four regulated reactions in the gluconeogenesis pathway (catalyzed by PC, PEPCK and G6Pase) were predicted to carry higher metabolic flux in cells expressing either basal or activated p53 than in any of the other states (Figure 2 and Additional file 1: Table S1). Moreover, the shuttling of glucose-6-phosphate (G6P) from the cytosol to the endoplasmic reticulum (where G6P is dephosphorylated by G6Pase), which is essential for completion of gluconeogenesis, was predicted to carry higher metabolic flux in p53-expressing cells than in HepG2^sh-p53^ (Figure 2 and Additional file 1: Table S1).

**Figure 3:**
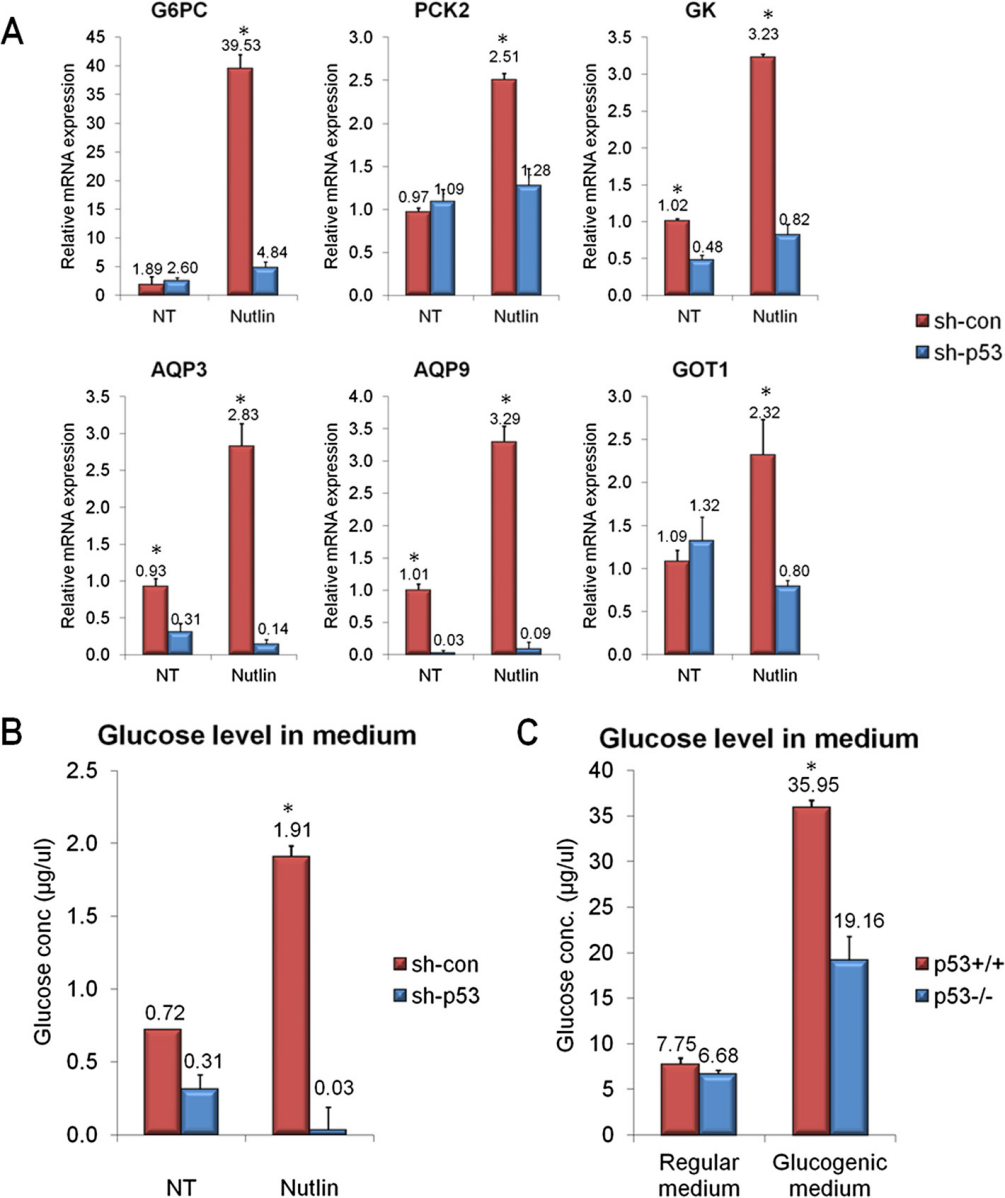
**p53 enhances gluconeogenesis-related gene expression and augments hepatic glucose production (HGP)**. (**A**) HepG2 cells were treated with the p53-activating agent Nutlin-3a (10μM) for 24 hours, total RNA was extracted and the mRNA levels of the indicated genes were analyzed in qPCR analysis (as described in [4]). The primers used for qPCR are described in Additional file 1. (**B**) Densely-plated HepG2 cells were incubated in glucogenic medium [15] for 24 hours. Glucose secretion to the media was measured by the glucose oxidase colorimetric method. (**C**) Hepatocytes were isolated and incubated in gluconeogenic medium [15] for 4 hours. Glucose secretion to the media was measured by the glucose oxidase colorimetric method. All presented data are representative experiments from at least three repeats. Each bar represents an average of three triplicates. Columns marked with asterisks denote a significant (*P* <0.05) elevation, measured by a student’s t-test compared to unmarked columns. qPCR, quantitative PCR.

In light of this prediction and in an attempt to find glucogenic genes whose expression is altered in the presence and activation of p53, we examined the expression levels of several key gluconeogenesis-related genes using quantitative PCR (qPCR). The mRNA levels of *G6PC* (encoding the catalytic unit of G6Pase) and *PCK2* (encoding mitochondrial PEPCK) were significantly elevated in Nutlin-treated HepG2^sh-con^ cells but not in Nutlin-treated HepG2^sh-p53^ cells (Figure 3A). Moreover, several genes whose encoded proteins participate in supplying glucogenic precursors were elevated following Nutlin-3a treatment exclusively in HepG2^sh-con^ (aquaporin 3 (AQP3), aquaporin 9 (AQP9), glycerol kinase (GK) and glutamic-oxaloacetic transaminase 1 (GOT1)). Notably, most of these genes participate in the uptake and metabolism of glycerol into the glucogenic pathway following lypolysis (AQP3, AQP9 and GK; Figure 2).

In accordance with our *in silico* predictions and with the p53-dependent changes in glucogenic gene expression, we found that HepG2 cells exhibit increased hepatic glucose production (HGP) following Nutlin-3a treatment (Figure 3B). Moreover, primary hepatocytes isolated from p53-deficient mice are impaired in their glucogenic capacity compared to wild-type mice (Figure 3C). In order to exclude any effects of cell viability affected by different p53 statuses on total glucose levels, we normalized the glucose level to total protein level yielding similar results (Additional file 3: Figure S1). To summarize, we found that p53 promotes the expression of genes whose encoded proteins are involved in both supplying glucogenic precursors and gluconeogenesis. Accordingly, p53 promotes glucose secretion in both human and mouse hepatocytes.

In combination with gene expression analysis, the iMAT flux-prediction method can infer directionality of reactions and metabolite production capacities. iMAT treats the expression levels of enzymes as cues for the likelihood that their associated reactions carry metabolic flux. It then uses network integration to accumulate these cues into a global, consistent prediction of metabolic behavior. As such, iMAT can reveal levels of post-transcriptional regulation that are not reflected in the gene expression data. Therefore, the proteins associated with reactions predicted to carry higher flux are not necessarily regulated at the expression level and may be either regulated at the level of translation or post-translationally. Moreover, the flux through a certain reaction is calculated not only as a function of gene expression and regulation, but also with respect to the sum of reactions in the cell and is therefore affected by changes in expression of proteins not directly associated with the reaction. Thus, iMAT is a reliable predictor of metabolic fluxes [10].

By employing iMAT, we were able to find a role for p53 in enhancing gluconeogenesis. Moreover, we found that p53 induces the expression of gluconeogenesis-related genes. As a sequence-specific transcription factor, p53 mostly regulates gene expression by binding to DNA regulatory elements and enhancing transcription. In order to examine the possibility that p53 directly regulates gluconeogenesis-related genes, we utilized published data employing chromatin immunoprecipitation of p53 followed by massive parallel sequencing (ChIP-seq) [16]. The p53 binding events nearest to the genes’ transcription start site (TSS) were several tens of kilobases away from the TSS and in most cases (excluding GOT1) these binding events were closer to other genes. This certainly does not exclude the possibility that p53 regulates these genes by direct DNA binding, since it is becoming evident that a transcription factor binding event (including in the case of p53) is not necessarily responsible for regulating the gene closest to it [17,18]. Moreover, the ChIP-seq data is from non-hepatic cells which are not glucogenic and the possibility exists that the chromatin around gluconeogenesis-related genes in non-glucogenic cells is inaccessible to transcription factor binding. Another plausible scenario is that p53 regulates gluconeogenesis-related genes in an indirect manner, namely through inducing the transcription or the activity of a glucogenic transcription factor. For example, p53 was found to augment the gene-inducing activity of a known glucogenic transcription factor, glucocorticoid receptor (GR), (although the authors inspected the effect of p53 on properties of GR that are not linked to gluconeogenesis) [19].

In accordance with the computational analysis and the gene expression data, we found that p53 enhances glucose production in both human and mouse hepatocytes. Our findings are strongly supported by several published data. First, p53 induces the expression of TP53-induced glycolysis and apoptosis regulator (TIGAR), which lowers the levels of fructose-2,6-bisphosphate (F2,6BP), an inhibitor of gluconeogenesis [20]. Second, p53 inhibits glucose-6-phosphate dehydrogenase (G6PD), an enzyme that metabolizes G6P in the pentose phosphate pathway (PPP) [21]. Thus, p53 may enhance gluconeogenesis by both increasing glucogenic flux due to lower F2,6BP (resulting in higher G6P levels) and by blocking the shunting of G6P to the PPP. Third, p53 forms a complex with peroxisome proliferator-activated receptor gamma coactivator 1 alpha (PGC1α), leading to modulation of p53-dependent transcription [22]. The authors also measured HGP as a general indicator of liver function. p53-deficient mice showed reduced glucogenic capacity compared with wild-type mice. PGC1α is an important coactivator of gluconeogenic transcription factors [23] and the possibility exists that PGC1α directly facilitates p53-dependent gluconeogenesis-related transcription, leading to the observed improved HGP in wild-type mice.

Reduced G6Pase levels are correlated to the development of hepatocellular carcinoma [24]. It has been postulated that blocking the efflux of glucose to the bloodstream leads to its shunting to other pathways which are considered pro-cancerous such as the PPP and glycolysis [25]. Thus, our finding that p53 induces *G6PC* expression and enhances HGP adds another layer for the metabolic regulation exerted by p53 in an effort to curtail carcinogenesis.

## Availability of supporting data

The microarray results were deposited in GEO Accession Number GSE30137.

## Abbreviations

AQP3: Aquaporin 3; AQP9: Aquaporin 9; CBM: Constraint-based modeling; ChIP-seq: Chromatin immunoprecipitation-sequencing; DHAP: Dihydroxyacetone phosphate; F1,6BPase: Fructose-1,6-bisphosphatase; F2,6BP: Fructose-2,6-bisphosphate; FDR: False discovery rate; G3P: Glyceraldehyde 3-phosphate; G6P: Glucose-6-phosphate; G6Pase: Glucose-6-phosphatase; GCPC: Glucose-6-phosphatase, catalytic subunit; G6PD: Glucose-6-phosphate dehydrogenase; GK: Glycerol kinase; GOT1: Glutamic-oxaloacetic transaminase 1; GR: Glucocorticoid receptor; sh-con: Non-relevant short hairpin RNA; sh-p53: Short hairpin RNA targeting p53; HGP: Hepatic glucose production; iMAT: Integrative metabolic analysis tool; OAA: Oxaloacetate; PC: Pyruvate carboxylase; PCK2: Phosphoenolpyruvate carboxykinase 2; PEP: Phosphoenolpyruvate; PEPCK: Phosphoenolpyruvate carboxykinase; PGC1α: Peroxisome proliferator-activated receptor gamma coactivator 1 alpha; PPP: Pentose phosphate pathway; qPCR: Quantitative PCR; TIGAR: TP53-induced glycolysis and apoptosis regulator; TSS: Transcription start site.

## Competing interests

The authors declare that they have no competing interests.

## Authors’ contributions

IG conceived the study, designed and performed the experiments and drafted the manuscript. KY performed the computational analysis. SM and NG designed and performed the experiments. ER performed the computational analysis and drafted the manuscript. VR designed the experiments and drafted the manuscript. All authors read and approved the final version of the manuscript.

## Supplementary Material

Additional file 1 Detailed methods. Click here for file

Additional file 2 iMAT analysis. Click here for file

Additional file 3 **Figure S1** Hepatocytes were isolated and incubated in gluconeogenic medium [15] for 4 hours. Glucose secretion to the media was measured by the glucose oxidase colorimetric method. The glucose concentration was then normalized to total protein levels. (TIFF 277 kb) Click here for file
