# Supplementary material for: p53 promotes the expression of gluconeogenesis-related genes and enhances hepatic glucose production
Source: Cancer Metab. 2013 Feb 4;1:9. doi: 10.1186/2049-3002-1-9 (PMC4178212; doi:10.1186/2049-3002-1-9)
Supplement: Additional file 1 — Detailed methods. [file 2049-3002-1-9-S1.pdf]

Constraint-based modeling:

iMAT accepts as input a set of highly and lowly expressed genes based on gene expression levels. Next, it looks for a consistent enzyme activity solution, where a maximal number of reactions that are considered highly expressed indeed carry metabolic flux and a maximal number of reactions that are considered lowly expressed don't carry any metabolic flux. Since our modeling technique takes into account other constrains such as thermodynamic and steady-state constrains, the solutions obtained by iMAT aims to capture modifications that go beyond the explicit gene expression information to capture post-transcriptional regulation effects. Under the maximal similarity to the gene expression measurements, we further perform two analyses:

1. For each reaction in the human metabolic model we examined its minimal and maximal allowed flux in each condition. This analysis allowed us to identify the set of reactions that is predicted to be confidently active in one condition and inactive in all other conditions.
2. iMAT's solution is not unique as a space of alternative optimal solutions may exist. Therefore, to go beyond the range of allowed fluxes computed in (1) and obtain an estimated flux rate for each reaction in the network, we sampled 100 different flux distributions that are all consistent with the reactions' state of activity or inactivity. The mean flux distribution obtained over these 100 samples then serves as an approximation of the flux carried by the relevant reactions.

Pathway enrichment analysis:

The pathway enrichment analysis was carried by a hypergeometric test where the background is the number of reaction found in the human model while excluding those that are considered dead-end (i.e., cannot carry a metabolic flux in the given condition). The overlap of each metabolic pathway with the set of active and inactive reactions (as defined above) is then examined via a simple hypergeometric test. All pathways achieving a significant P-value after correcting for multiple hypothesis using FDR with $\alpha = 0.05$ appear in the file *Supplemental Table 1.xlx*.

Primers used for quantitative PCR:

Gene symbol	Primer Details
G6PC	Forward: CGACCTACAGATTTCGGTGCTT<br>Reverse: ATGAGGAAAATGAGCAGCAAGG
PCK2	Forward: GGCTGAGAATACTGCCACACT<br>Reverse: ACCGTCTTGCTCTCTACTCGT
GK	Forward: CATGGCAGCCTCAAAGAAGG<br>Reverse: AAAACCAAAAAGCGCGTCG
AQP3	Forward: GGGGAGATGCTCCACATCC<br>Reverse: AAAGGCCAGGTTGATGGTGAG
AQP9	Forward: TTCAATGGCAGTTGCAATGG<br>Reverse: TTCATCCGTCCAAAGAGACACA
GOT1	Forward: ATTTCTTAGCGCGTTGGTACA<br>Reverse: ACACAGCATTGTGATTCTCCC
