# Supplementary figures and images for: p53 promotes the expression of gluconeogenesis-related genes and enhances hepatic glucose production
Source: Cancer Metab. 2013 Feb 4;1:9. doi: 10.1186/2049-3002-1-9 (PMC4178212; doi:10.1186/2049-3002-1-9)

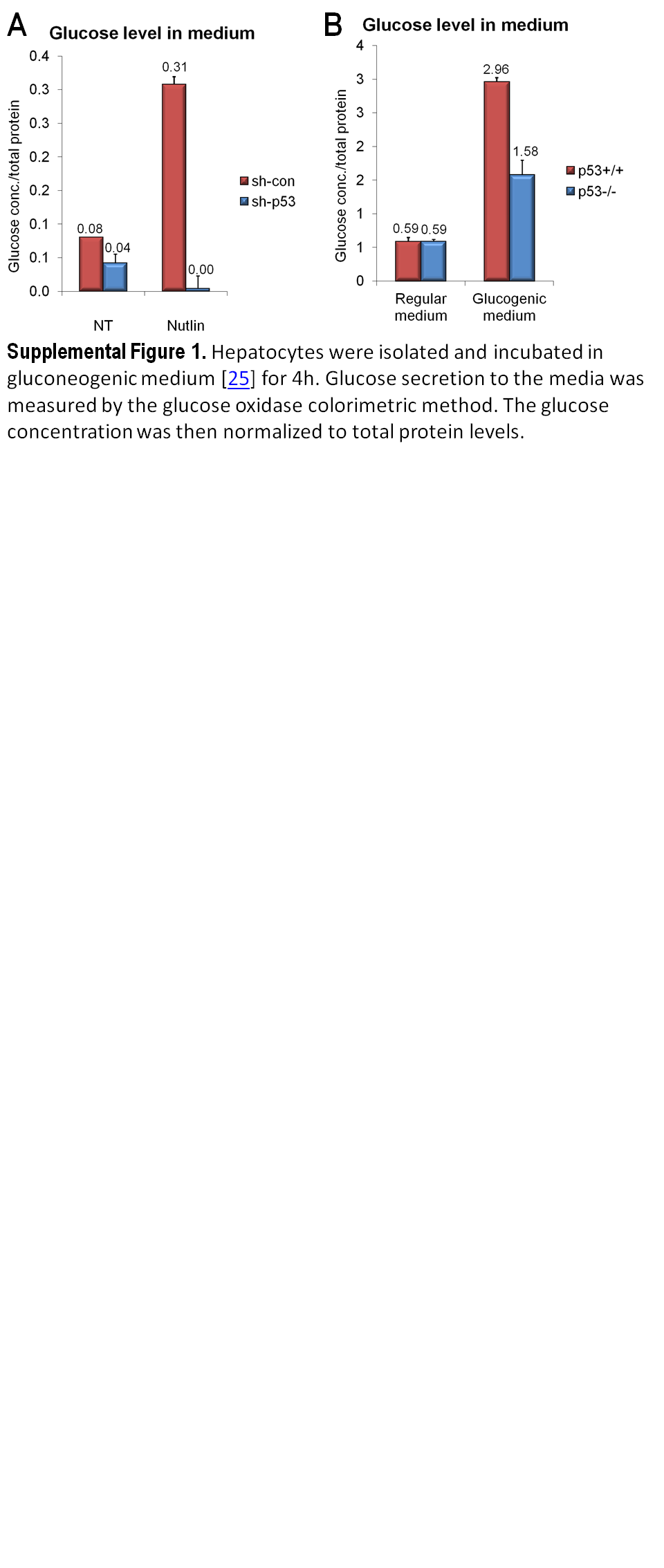

Supplement: Additional file 3 — Figure S1 Hepatocytes were isolated and incubated in gluconeogenic medium [15] for 4 hours. Glucose secretion to the media was measured by the glucose oxidase colorimetric method. The glucose concentration was then normalized to total protein levels. (TIFF 277 kb) [file 2049-3002-1-9-S3.tiff]
